# Identification of a Divergent Lineage Porcine Pestivirus in Nursing Piglets with Congenital Tremors and Reproduction of Disease following Experimental Inoculation

**DOI:** 10.1371/journal.pone.0150104

**Published:** 2016-02-24

**Authors:** Bailey L. Arruda, Paulo H. Arruda, Drew R. Magstadt, Kent J. Schwartz, Tyler Dohlman, Jennifer A. Schleining, Abby R. Patterson, Callie A. Visek, Joseph G. Victoria

**Affiliations:** 1 Department of Veterinary Diagnostic and Production Animal Medicine, College of Veterinary Medicine, Iowa State University, Ames, Iowa, United States of America; 2 Boehringer Ingelheim Vetmedica, Inc., Ames, Iowa, United States of America; University of Hong Kong, CHINA

## Abstract

Congenital tremors is a sporadic disease of neonatal pigs characterized by action-related repetitive myoclonus. A majority of outbreaks of congenital tremors have been attributed to an unidentified virus. The objectives of this project were to 1) detect potential pathogen(s) in samples from piglets with congenital tremors and 2) develop an infection model to reproduce disease. Using next-generation sequencing, a divergent lineage pestivirus was detected in piglets with congenital tremors. The virus was originally most closely related to a bat pestivirus but is now more closely related to a recently published novel porcine pestivirus provisionally named atypical porcine pestivirus. A quantitative real-time PCR detected the virus in samples from neonatal piglets with congenital tremors from two separate farms, but not in samples from unaffected piglets from the same farm. To fulfill the second objective, pregnant sows were inoculated with either serum containing the pestivirus or PBS (control) by intravenous and intranasal routes simultaneously with direct inoculation of fetal amniotic vesicles by ultrasound-guided surgical technique. Inoculations were performed at either 45 or 62 days of gestation. All sows inoculated with the novel pestivirus farrowed piglets affected with congenital tremors while PBS-inoculated control piglets were unaffected. Tremor severity for each piglet was scored from videos taken 0, 1 and 2 days post-farrowing. Tremor severity remained relatively constant from 0 to 2 days post-farrowing for a majority of piglets. The prevalence of congenital tremors in pestivirus-inoculated litters ranged from 57% (4 out of 7 affected piglets) to 100% (10 out of 10 affected piglets). The virus was consistently detected by PCR in tissues from piglets with congenital tremors but was not detected in control piglets. Samples positive by PCR in greater than 90% of piglets sampled included brainstem (37 out of 41), mesenteric lymph node (37 out of 41), tracheobronchial lymph node (37 out of 41), and whole blood (19 out of 20). Although the first description of congenital tremors was in 1922, this is the first reported reproduction of congenital tremors following experimental inoculation with a divergent lineage porcine pestivirus. Studies investigating disease mechanism, epidemiology, and diagnostic assay development are needed to better understand the pathophysiology of congenital tremors due to this pestivirus.

## Introduction

Congenital tremors (CT; *myoclonia congenita*) is a disease of neonatal pigs that is characterized by bilateral, clonic contractions of skeletal muscle that are observed within hours of birth and cease when piglets are at rest [1]. Tremors vary in intensity from mild with only a fine tremor being evident in the head, flank or hind leg region, to prominent repetitive contractions that make it difficult for the piglet to stand or walk (S1 MP4) [1]. Although clinical signs vary in severity and severity is not thought to be progressive, the survival of severely affected piglets may be threatened due to nursing difficulties, and deaths can result from starvation and inadequate colostrum intake [1]. Early reports of the disease were made by Kinsley (1922) in the United States [2], Payen and Fournier (1934) in France [3], and Hindmarsh (1937) in Australia [4]. Although sporadic, the disease is widely distributed and has been reported in several European countries, Australia, New Zealand, North America, South America and likely occurs globally [1].

The term CT defines the clinical signs that may be observed in fetal infections with classical swine fever virus (CSFV; Type AI) [5], inherited disorders (Type AIII and Type AIV) [6,7], and trichlorfon toxicity (Type AV) [8]. Based on the elimination of CSFV; genetic variability in swine herds; and current production practices, a majority of outbreaks of congenital tremors have been attributed to an unidentified virus (Type AII) [1,9].

Congenital tremors Type AII has no apparent breed predilection [1]. Disease is more prevalent in litters of gilts than sows [10], suggesting that the immune status of the dam is important in the development of disease in the piglet. Morbidity in outbreaks of CT varies both within and between litters; a few pigs in one or two litters or all pigs in several litters may be affected [1]. Outbreaks typically occur in several litters farrowed over a 1-week to 2-month period [1]. Adjacent farms usually remain disease-free and after the initial outbreak, the disease rarely recurs in subsequent litters from a sow [1].

To investigate the cause of CT Type AII, the objectives of the study described here were twofold: 1) identification of an infectious etiology in piglets with congenital tremors using next-generation sequencing and 2) reproduction of CT following experimental challenge with the infectious agent using a novel challenge model.

## Materials and Methods

### Pestivirus Identification

#### Next-generation sequencing

Varied porcine tissues (serum, cerebrum, cerebellum, spinal cord, cerebrospinal fluid (CSF), and/or lung) from three diagnostic investigations of CT were obtained: lung tissue from a single piglet (ID 20130103); either pooled brain tissue or pooled lung tissue from six piglets (ID 20120705); and CSF (n = 2; Farm B), serum (n = 2; Farm A and B), and lung (n = 2; Farm A and B) from six different piglets originating from two different farms (ID 2014016573). With the exception of the lung tissue from sample ID 20120705, all samples tested exhibited at least partial pestivirus genomic sequence. Serum or tissue homogenates were re-suspended in Hanks balanced salt solution (Corning-Cellgro) and enriched for viral particle protected nucleic acids by digestion with a combination of nucleases: RNase A (Invitrogen), Baseline Zero DNase (Epicentre), and Turbo DNase (Invitrogen) as described previously [11]. Viral nucleic acids were extracted per the manufacturer’s protocol using Qiagen Viral RNA blood kit. Post-extraction, nucleic acids were further treated with Turbo DNase to remove host or potential viral DNA, thus further enriching for viral RNA. Double-stranded cDNA was generated through reverse transcription and Klenow (NEB) treatment using priming with random hexamers as described previously [11].

Samples were processed for MiSeq based sequencing through library generation using the NextEra XT library preparation kit (Illumina) per the manufacturer’s suggested protocol, with replacement of column elution (Qiagen, MinElute) in lieu of bead normalization. The library was run on the MiSeq using the 500-cycle kit (Illumina) and data was analyzed using a combination of NextGene (version 2.3.4.2) and Sequencher software (version 5.1). High quality sequences were selected as those containing a median Q-score of greater than 25 and trimmed with a cut-off of no more than 3 uncalled bases at 3’-end or 3-consecutive bases with Q-score measuring less than 16. *De novo* assembled sequences were analyzed by comparison to GenBank sequence via BLASTn and BLASTx. ClustalW alignment was used for phylogenetic analysis of the 215 amino acid sequence of of the NS3 gene and 170 amino acid sequence of the Npro gene. Neighbor-joining phylogenetic trees were generated from 1,000 replicates using MEGA 6.0 software.

#### Quantitative real-time polymerase chain reaction (RT-qPCR)

A RT-qPCR targeting the N3S region of the genome of the divergent lineage pestivirus was designed. Tissues samples (n = 362) from growing pigs that were submitted to the Iowa State University Veterinary Diagnostic Laboratory (ISU VDL) for routine diagnostic testing were used to determine the frequency of the pestivirus in this sample set. Two sample sets were also collected from farms with congenital tremors. These samples included serum, cerebrum, cerebellum, brainstem, and spinal cord. The first set (Farm A) consisted of 6 affected and 2 unaffected pre-suckle piglets, serum from five sows from which the pre-suckle piglets were selected, and 5 affected and 2 unaffected post-suckle piglets between 6- and 14-days-old. The second set (Farm B: ISUVDL2014016573) consisted of 5 affected piglets suckle status unknown and serum from five sows with affected piglets.

The quantitative one-step RT-PCR kit (iTaq Universal Probes One-Step Kit; BioRad, cat no. 172–5141) was carried out in a 25μl reaction containing 2μl of extracted total nucleic acid, 1.0μl of probe (2μM), 1μl of each primer (5μM), 12.5μl of 2X RT-PCR mix, 0.5μl iScript reverse transcriptase and 7.0μl of DEPC-treated water (Table 1). The reaction took place using a CFX96 real-time PCR detection system (BioRad) under the following conditions: initial reverse transcription at 50°C for 10min, followed by initial denaturation at 95°C for 3 min, 40 cycles of denaturation at 95°C for 15s and annealing and extension at 57°C for 30s. To generate quantitative data, a pestivirus ultramer was included in each run (Integrated DNA Technologies) encompassing the NS3 region targeted by the primers. A cut-off for positive samples was established at cycle quantification (Cq) values lower than 36.

**Table 1:** Real-time PCR Primer, Probe and Ultramer Sequences.

	Sequence
Pesti_6332_F	TGC CTG GTA TTC GTG GC
Pesti_6455_R	TCA TCC CAT GTT CCA GAG T
Pesti_6351_P	/5Cy5/CCT CCG TCT CCG CGG CTT CTT TGG /3BHQ_2/
Pesti_ultra	AAC AGG AAA GAA CTG CCT GGT ATT CGT GGC AAC CAA AGA AGC CGC GGA GAC GGA GGC TAA AGA ACT GCG CAC CAG AGG AAT TAA CGC CAC CTA CTA CTA TTC AGG TAT AGA CCC TAA GAC TCT GGA ACA TGG GAT GAC CAA TCA GCC AT

### Sow Inoculation Model

#### Animals

All procedures were approved by the Institutional Animal Care and Use Committee of Iowa State University (Log Number: 1-14-7907-S 2). Eight individually identified crossbred sows at 38 days of gestation were obtained from a commercial source with no known previous history of CT. Serum from all sows was negative for PCV2a, PCV2b, PRRSV, PPV1, PPV5 and the novel pestivirus by RT-qPCR prior to shipment and inoculation. Individual sows were randomly assigned to one of three groups housed separately [sham-inoculated at 45 days gestation (n = 1) and 62 days gestation (n = 1), pestivirus-inoculated at 45 days gestations (n = 3), and pestivirus-inoculated at 62 days gestation (n = 3)] and were fed a nutritionally complete diet throughout the study period.

#### Animal inoculation

Sows were held off feed and water for 12 hours prior to surgery to reduce the risk of anesthetic regurgitation. Terminal serum from a viremic pig (ISUVDL2014016573) was thawed at 37°C. Total nucleic acid was extracted and screened by PCR for the presence of PCV2a, PCV2b, PRRSV, PPV1, PPV5 and the pestivirus; only the pestivirus was detected (Cq = 27.47). Serum was 0.2μm filtered and diluted by adding 6mL of sera to 35mL of 1X PBS (Gibco). On the day of inoculation, inoculum was thawed and held on ice during the inoculation procedure. General anesthesia was induced with an intramuscular injection of a combination of tiletamine and zolazepam (Telazol^®^), ketamine, and xylazine. Following anesthetic induction, each sow was placed in left lateral recumbency, and the right abdomen prepared for aseptic laparotomy. The abdomen was draped for surgery and a local line block with 2% lidocaine was administered prior to incision. An approximately 30cm paramedian incision was made ~5cm lateral to the mammary tissue to gain access to the abdominal cavity. The uterus was exteriorized and a sterile handheld linear array ultrasound transducer was used to image each fetal unit and guide the inoculation needle into the fetal amniotic vesicle. Each vesicle was inoculated with 0.25mL of inoculum (PBS or pestivirus-serum) using a small gauge needle (22g) (S2 MP4). The abdominal wall was closed in three layers using size 2 polyglactin 910 suture. The inoculum was also administered directly to the sow via an intranasal (2mL) and intravenous (2mL) route immediately following the surgical procedure. Single doses of flunixin meglumine (Banamine-S^®^) and ceftiofur crystalline free acid (Excede^®^) were given intramuscularly immediately after incisional closure and prior to anesthetic recovery. Anesthetic induction occurred at 8:30 AM for the first sow on the respective day of surgery. Each procedure took approximately 1 hr. The anesthetic induction of the final sow occurred at 11:30 AM.

#### Clinical observations, sample collection, and necropsy

After inoculation, sows were monitored daily and rectal temperatures were taken from 0–7 days post-inoculation (DPI). Fecal material, blood and nasal swabs were collected from sows at DPI 2, 7, 10 and 14 and then weekly until farrowing. At the time of farrowing, piglets were individually identified and serum, nasal swabs and fecal swabs were collected. In a subset of piglets (n = 7), blood from the umbilical cord was collected. Videos of individual piglets were taken daily from 0–2 days post-farrowing (DPF). Four investigators blinded to groups reviewed the videos and each piglet received a tremor severity score: 0 –absent, 1 –fine muscle fasciculation, 2 –mild tremor, 3 –moderate tremor, 4 –severe tremor with pronounced hopping. Scores were then averaged to assign each piglet an overall tremor severity score by DPF. Piglets receiving a score of ≥ 0.75 on DPF 2 were considered to be affected. The presence or absence of splay leg was also recorded on each DPF for each piglet. Sows and piglets were euthanized on DPF 2 via captive bolt gun and injectable barbiturate overdose, respectively. At necropsy piglet serum, cerebrum, cerebellum, brainstem, spinal cord, kidney, mesenteric lymph node, tracheobronchial lymph node, thymus, heart, and spleen were collected. In a subset of piglets, whole blood (EDTA tubes; n = 20) and CSF (n = 29) were collected. Sow serum was also collected at necropsy.

## Results

### Pestivirus Identification

#### Next-generation sequencing

**Fig 1:**
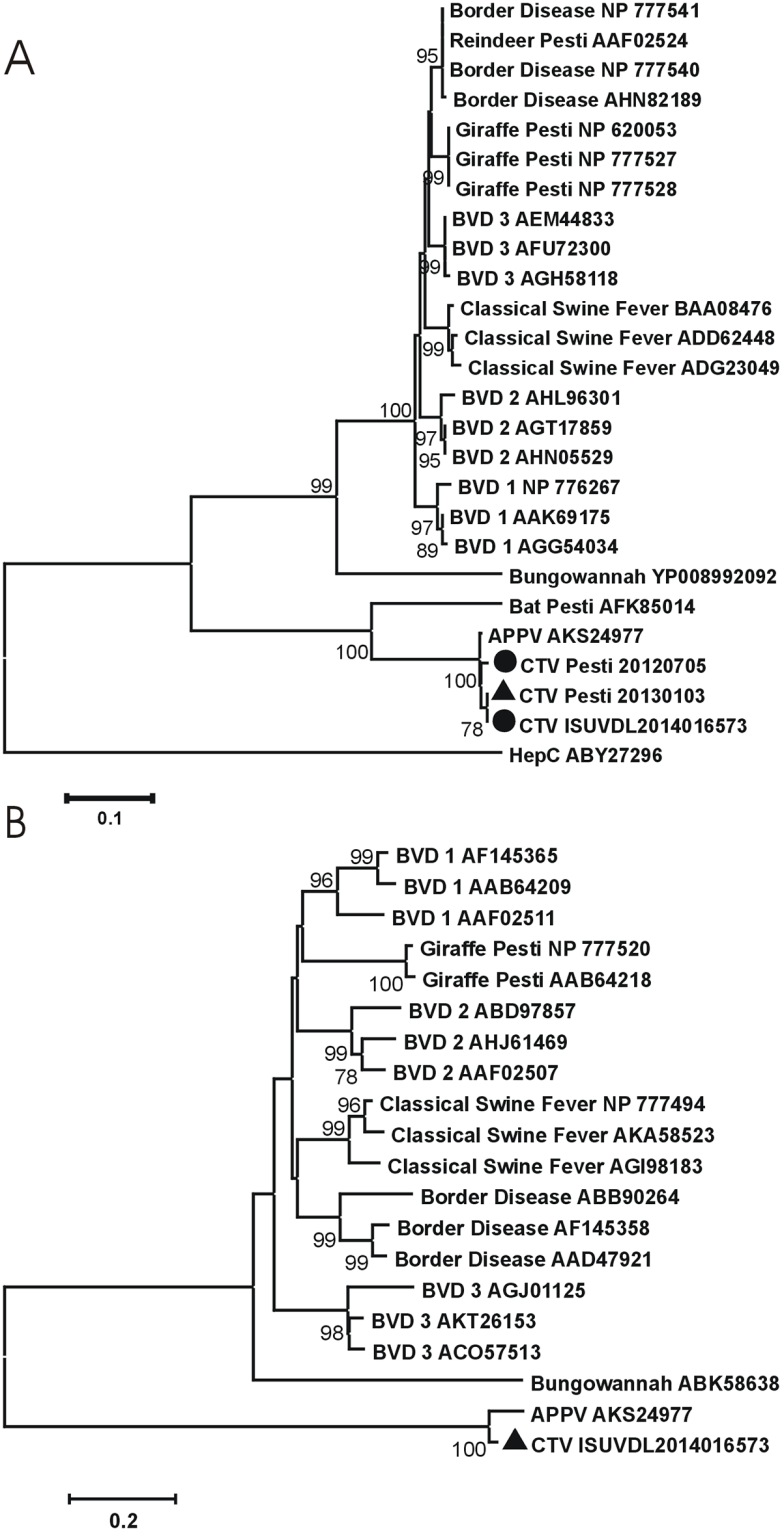
Phylogenetic association of pestiviruses. Neighbor-joining phylogenetic trees generated with 1,000 bootstrap samplings (MEGA 6.0) for pestivirus NS3 (A) and Npro (B) amino acids aligned by ClustalW multiple alignment. GenBank accession numbers for each sample indicated in name. Circles indicate sequences described from this study and triangle indicates the sequence from the virus described in this study used for inoculation.

Through the use of next-generation sequence technology a virus closely related to a Chinese bat pestivirus, and now known to be more closely related to a recently reported provisionally named atypical porcine pestivirus was discovered from three independent congenital tremor disease investigations. The near-complete genome was obtained from one of the three investigations. This virus in the serum from a viremic animal was subsequently used for animal inoculations in this study. Phylogenetic analysis of the NS3 and Npro support classification of the virus identified herein as a member of the putative “atypical porcine pestivirus” species (Fig 1), with 88.0% and 94.6% nucleotide and amino acid identity, respectively. A retrospective analysis of pestivirus RNA by RT-qPCR from cases submitted to the ISU VDL indicated 21 of 362 samples (6%) were positive. These cases were routine submissions from herds experiencing varied clinical signs.

#### RT-qPCR

Piglet samples from animals exhibiting congenital tremors and unaffected cohorts were collected from two farms, Farm A and Farm B. Animals that were diagnosed with congenital tremors were positive for the pestivirus by RT-qPCR while the virus was not detected in the central nervous tissue or serum of unaffected piglets (Table 2). The virus was detected in the serum from a single sow from Farm A (S1 Table).

**Table 2:** Quantitative Real-time PCR Results from Piglet Samples from Farm A and Farm B.

			Sample Type									
			*Cerebrum*		*Cerebellum*		*Brainstem*		*Spinal Cord*		*Serum*	
*Farm*	*Animal ID*	*Disease Status*^a^	Cq^b^	SQ^c^	Cq	SQ	Cq	SQ	Cq	SQ	Cq	SQ
	P1	-	U^d^	0	U	0	U	0	U	0	U	0
	P2a	+	U	0	34.18	3.95E+02	35.93	1.36E+02	33.39	6.38E+02	30.64	1.14E+05
	P2b	+	U	0	35.92	1.37E+02	U	0	35.53	1.74E+02	30.14	1.47E+05
	P4a	+	U	0	32.44	1.13E+03	U	0	36.51	9.56E+01	36.44	6.62E+03
	P4b	+	U	0	29.37	2.14E+05	35.41	1.87E+02	U	0	30.97	9.71E+04
	P5a	-	U	0	U	0	U	0	U	0	U	0
	P6a	+	U	0	33.65	4.76E+02	U	0	33.89	4.71E+02	U	0
A	P6b	+	U	0	28.75	2.89E+05	U	0	U	0	31.37	8.00E+04
	1	+	32.65	1.00E+03	U	0	U	0	35.65	1.61E+02	30.92	1.05E+05
	2	+	U	0	32.31	1.23E+05	U	0	35.72	1.54E+02	30.77	1.13E+05
	3	-	U	0	U	0	U	0	U	0	U	0
	4	-	U	0	U	0	U	0	U	0	U	0
	5	+	U	0	30.50	3.69E+03	U	0	35.90	1.38E+02	33.97	2.31E+04
	6	+	ND^e^	ND	ND	ND	ND	ND	ND	ND	29.40	2.23E+05
	7	+	U	0	32.39	0	U	0	U	0	31.29	8.74E+04
	20	+	26.59	8.36E+05	24.04	2.92E+06	24.56	2.27E+06	25.50	1.42E+06	26.04	1.09E+06
	21	+	30.92	9.96E+04	26.25	9.89E+05	27.41	5.58E+05	26.14	1.04E+06	22.26	6.98E+06
B	22	+	25.79	1.24E+05	29.32	2.19E+05	27.31	5.85E+05	26.14	1.04E+06	22.25	7.04E+06
	23	+	27.51	5.31E+05	23.45	3.91E+06	26.43	9.05E+05	24.46	2.38E+06	22.47	6.31E+06
	24	+	27.93	4.34E+05	24.13	2.79E+06	27.25	6.05E+05	24.10	2.38E+06	22.25	7.04E+06

^a^Presence (+) or absence (-) of congenital tremors.

^b^Cq = quantification cycle value.

^c^SQ = starting quantity.

^d^U = “undetected” following 40 cycles.

^e^ND = Not done.

### Sow Inoculation Model

#### Sow observations and samples

One sham-inoculated sow at 45 days gestation developed a moderate fever following surgery and aborted all fetuses on DPI 3 and 4. A sow from the group to be inoculated at 45 days of gestation was found not to be pregnant at time of inoculation; she was removed from the study. Sham-inoculated and pestivirus-inoculated sows did not display clinical signs nor did they develop a detectable viremia or shed the virus at levels detectable by RT-qPCR. All sows farrowed naturally. There was one stillborn piglet (Sow ID 3661) and one macerated fetus (Sow ID 3500).

#### Piglet Observations and Samples

Sham-inoculated piglets did not have clinical signs consistent with CT on DPF 0, 1, or 2 (S4 MP4). A majority of piglets that were pestivirus-inoculated as fetuses at 45 or 62 days gestation had clinical signs consistent with CT (S4 MP4). The prevalence of congenital tremors (S5 MP4) and splay leg (S6 MP4) in pestivirus-inoculated litters ranged from 57% to 100% and 0% to 40% on DPF 2, respectively (Table 3). Tremor severity varied within litters by piglet but remained relatively constant over the 2 day observation period in a majority of piglets (Table 4).

**Table 3:** Prevalence of Congenital Tremors and Splay Leg in Pestivirus-Inoculated Litters on Day 2 Post-farrowing.

	Congenital Tremors		Splay Leg	
*Sow ID/Gestation Day*^a^	*No*. *Affected*^b^ */ No*. *in Litter*	*Prevalence (%)*	*No*. *Affected / No*. *in Litter*	*Prevalence (%)*
4036/45	5 / 8	62.5	1 / 8	12.5
3992/45	7 / 9	77.7	2 / 9	22.2
3661/62	4 / 6	66.6	0 / 6	0.0
3500/62	10 / 10	100	4 / 10	40.0
4023/62	4 / 7	57.1	0 / 7	0.0

^a^Day of gestation at time of inoculation.

^b^Piglets were considered to be affected by congenital tremors if the tremor severity score was ≥ 0.75.

**Table 4:** Congenital Tremor Score by Piglet and Days Post-Farrowing.

		Average Tremor Severity Score		
*Sow ID/Inoculum/ Gestation Day*^a^	*Animal ID*	*DPF*^b^ *0*	*DPF 1*	*DPF 2*
	71	0	0	0
	72	0.25	0	NA
	73	0	0	0
2427/PBS/62	74	0.50	0	0
	75	0	0	0
	124	0.25	0.5	0
	125	0	0	0
	31	2.00	0	0.75
	32	0.25	0.25	0
	33	3.50	4.00	4.00
4036/pestivirus/45	34	0.50	0	0
	35	3.75	4.0	4.0
	36	3.75	4.0	4.0
	37	1.00	0	0.25
	38	3.50	3.5	3.5
	40	4.00	3.25	3.25
	41	0.25	0	0
	42	3.00	1.75	1.5
	43	2.00	0.25	0.25
3992/pestivirus/45	44	2.50	1.50	1.75
	45	3.00	3.75	4.00
	46	3.25	2.50	2.75
	47	2.25	1.25	1.25
	48	3.00	2.00	2.50
	94	1.00	2.5	3.0
	95	0	NA	NA
	96	2.00	3.00	3.25
3661/pestivirus/62	97	0.75	0	0
	98	2.50	2.0	2.5
	99	2.25	2.50	2.25
	100	0	0	0.25
	89	2.75	2.75	3.25
	90	3.75	3.25	3.50
	111	3.50	3.00	2.50
	112	1.75	NA	NA
	113	2.50	2.50	3.00
3500/pestivirus/62	116	3.25	3.75	4.00
	117	3.50	3.25	3.25
	118	3.25	4.00	3.75
	121	2.75	1.75	3.00
	122	2.00	2.75	2.75
	123	3.00	2.75	2.75
	114	0.50	0	0.50
	115	1.50	3.50	4.00
	119	1.00	1.50	2.25
4023/pestivirus/62	120	0	0	0.25
	130	1.00	0.50	2.25
	131	0	1.00	0
	132	1.75	0.25	0.75

^a^Day of gestation at time of inoculation.

^b^DPF = Days post-farrowing.

**Fig 2:**
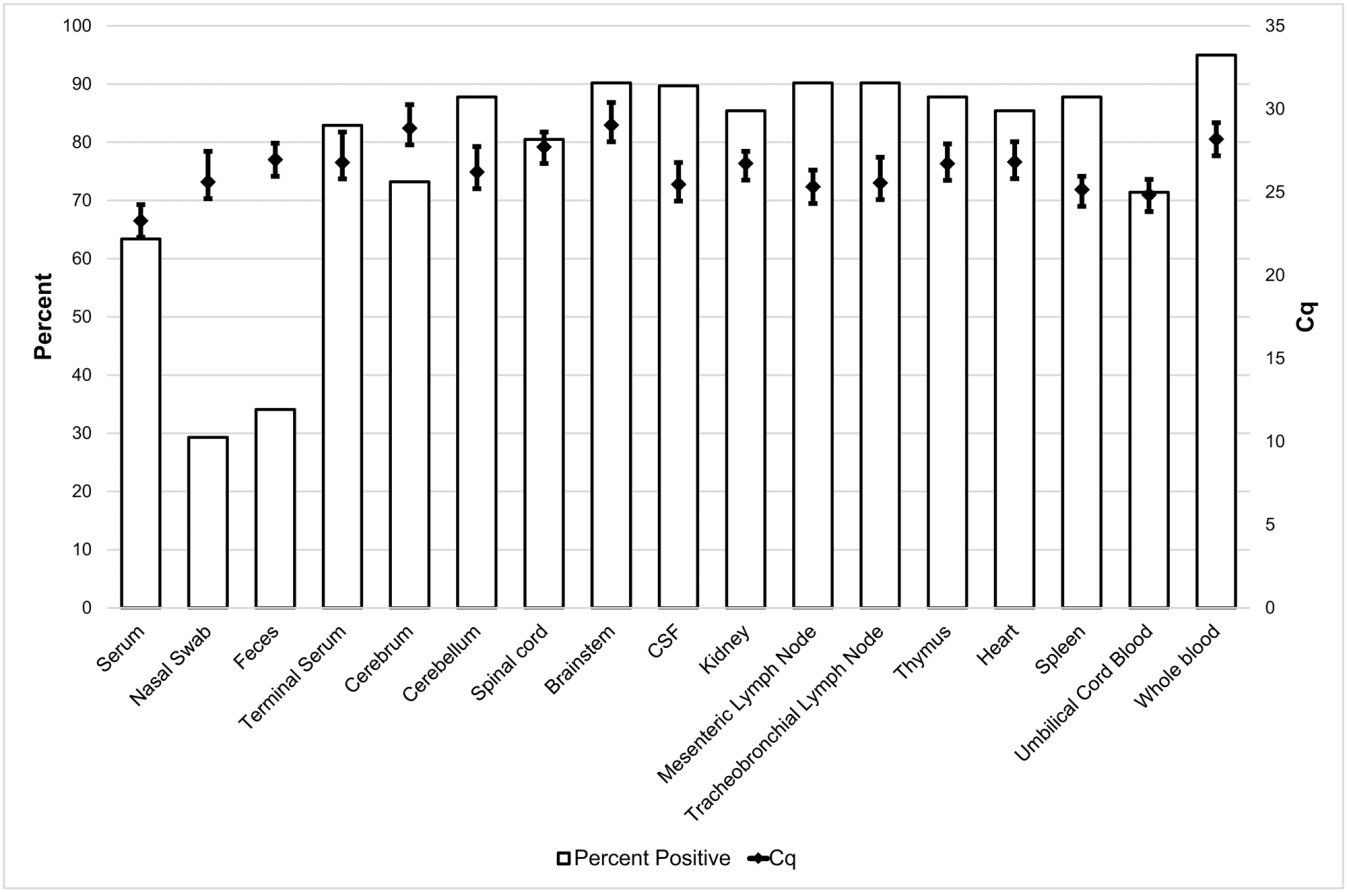
Percent Positive and Average RT-qPCR Cq by Sample Type. Pestivirus RNA detected by RT-qPCR targeting the NS3 gene. Viral RNA was not detected in PBS-inoculated piglets.

Viral RNA was detected in all piglets with CT. Viral RNA was also detected in ten out of eleven pestivirus-inoculated unaffected piglets. Pestivirus RNA was detected in serum (26 out of 41), nasal swabs (12 out of 41), feces (14 out of 41), terminal serum (34 out of 41), cerebrum (30 out of 41), cerebellum (36 out of 41), brainstem (37 out of 41), spinal cord (33 out of 41), kidney (35 out of 41), mesenteric lymph node (37 out of 41), tracheobronchial lymph node (36 out of 41), thymus (37 out of 41), heart (35 out of 41), and spleen (37 out of 41) by RT-qPCR in live-born pestivirus-inoculated piglets (Fig 2); viral RNA was not detected in the same samples from PBS-inoculated piglets (S2 Table). In addition, pestivirus RNA was detected in umbilical cord blood (5 out of 7), whole blood (19 out of 20), and CSF (26 out of 29) from a subset of piglets (Fig 2). The average Cq of serum, nasal swabs, CSF, mesenteric lymph node, tracheobronchial lymph node, spleen and umbilical cord blood was less than 26. The average Cq of feces, terminal serum, cerebellum, spinal cord, kidney, thymus, and heart ranged from 26 to 28. Cerebrum, brainstem, and whole blood had the highest average Cq values (>28). Pestivirus RNA was detected most commonly (>90% of the samples taken) in the brainstem, mesenteric lymph node, tracheobronchial lymph node, and whole blood; less commonly (80 to 90% of the samples taken) in terminal serum, cerebellum, spinal cord, CSF, kidney, thymus, heart, and spleen; and least commonly (29 to 74% of the samples taken) in serum, nasal secretions, feces, cerebrum, and umbilical cord blood. Serum from two animals (35 and 90) were randomly selected to assess genomic stability by complete genome sequencing. Both animals exhibited identical 7 nucleotide fixed changes from the parental strain leading to four conserved amino acid changes (data not shown). Upon review of the deep sequencing data of the challenge material, evidence of polymorphism was observed at each of these positions (data not shown).

## Discussion

The syndrome of CT was first documented nearly 100 years ago; yet, most contemporary outbreaks have been attributed to an unidentified virus [1]. Using next-generation sequencing, a novel agent originally identified to be closely related to a bat pestivirus and now more closely related to a recently published divergent pestivirus provisionally named atypical porcine pestivirus [12] was detected in samples of piglets with CT.

A RT-qPCR was designed targeting the N3S portion of the genome of the divergent lineage pestivirus in order to detect viral RNA in multiple and varied sample types. A retrospective analysis detected pestivirus RNA by RT-qPCR in 6% (21 of 362) of samples from herds experiencing varied clinical signs suggesting that the virus is present in tissues from this sample set at a low prevalence. Samples from the inoculation study were selected based on clinical signs of CT and tissue distribution and replication sites of CSFV [13]. Tissue samples from piglets with CT from two unrelated farms contained viral RNA that was consistently detected in serum and central nervous system tissue suggesting that the virus has a systemic distribution while clinically impacting central nervous system function. This is further supported by the tissue distribution of viral RNA in the pestivirus-inoculated piglets. A specific site of replication was not determined, as all tested tissues had similar levels of detectable pestivirus RNA. This may suggest that viral replication occurs systemically and may include peripheral blood mononuclear cells or endothelial cells similar to CSFV [13].

The pestivirus used for this inoculation model was viremic serum as attempts at *in vitro* virus cultivation have not been successful. The immune status of the sows in this study is not known due to the lack of a serologic assay for this newly discovered virus. To avoid possible interference from anti-pestivirus antibodies in the sow, fetal amniotic vesicles were directly inoculated, as the porcine placenta does not allow the transfer of antibodies from the dam to the fetuses.

Although one PBS-inoculated sow aborted as a result of the surgical procedure, no clinical differences were observed between sham- and pestivirus-inoculated sows. Stillbirths, mummified or macerated fetuses have not been previously reported with CT outbreaks. The single stillbirth in one litter and single macerated fetus in another litter from pestivirus-inoculated sows were considered incidental and likely not a result of fetal infection. Despite IN and IV inoculation, sows did not develop a detectable viremia or shed the virus at levels detectable by RT-qPCR. Therefore, either the sows were not infected following challenge or the available diagnostic tests were insufficient to detect infection.

For CT to be manifested, it is likely that fetal infection must occur prior to development of fetal immunocompetence which occurs around 70–80 days of gestation in piglets [14]. In this study, fetuses at both 45 and 62 days of gestation were susceptible to infection with the divergent lineage pestivirus which resulted in CT in a majority of infected piglets. The selection of these two gestation time points was based on an approximate viremia of this pestivirus based on CSFV occurring prior to the development of fetal immunity (day 45 of gestation) and the development of the fetal central nervous system (day 62 of gestation) [15,16,17]. *In utero* pestivirus infections in other species at different gestational time points have differing clinical outcomes including reproductive failure, congenital malformations or immunotolerance whereby a persistently infected animal may shed virus throughout their lifetime [18]. In this study a number of pestivirus-inoculated piglets were born with splay leg. This condition is commonly observed in pigs; however, the pathogenesis and etiologies are currently speculative [19]. The role, if any, of this pestivirus in splay leg, reproductive failure in sows or ability for *in utero* infection to result in persistently infected animals requires additional investigation.

Overall, the clinical disease reproduced herein mimics naturally occurring outbreaks with variation in the prevalence of CT between litters and severity of clinical signs within litters. Viral RNA was detected in all piglets with CT. Moreover, viral RNA was detected in 41 out of 42 live-born pestivirus-inoculated piglets. Of the live-born pestivirus-inoculated piglets, eleven did not have CT on DPF 2 or DPF 0 (95), and viral RNA was detected in all pestivirus-inoculated unaffected piglets but one (95). Yet, the mechanism of central nervous systemic dysfunction in a majority of piglets but not all infected piglets is currently unknown. The ecology and pathogenesis of the host-virus interaction is undefined at this point but intriguing. Investigation of the role of persistent infection or dysfunctional immune response in clinical expression of CT and mechanism of central nervous system dysfunction is warranted. Literature concerning the mechanisms of tremor disorders in humans and animals is limited despite the high prevalence and importance of such symptomatology in human and veterinary medicine [20,21].

This study identified a recently described divergent porcine pestivirus in piglets with CT and not in unaffected cohorts and used this virus to reproduce CT through the development of an innovative inoculation technique. The successful development of virus isolation techniques, specific antibody assays, *in situ* detection techniques and refined molecular tools will undoubtedly lead to better understanding of pathogenesis and epidemiology of this virus.

## Supporting Information

S1 MP4 Piglet with Congenital Tremors. (MP4)

S2 MP4 Ultrasound-Guided Inoculation. (MP4)

S3 MP4 Neonatal Control Piglets at 2 Days Post-Farrowing. (MP4)

S4 MP4 Pestivirus-inoculated Piglets at 2 Days Post-Farrowing. (MP4)

S5 MP4 Tremor Severity Score Examples at 2 Days Post-Farrowing. (MP4)

S6 MP4 Piglet with Splay Leg. (MP4)

S1 Table Sow Serum RT-qPCR Results. The pestivirus was detected by RT-qPCR targeting the NS3 gene in the serum from a single sow that had farrowed piglets with congenital tremors.
(DOCX)

S2 Table RT-qPCR Results by Piglet and Sample Type. ^**a**^ Detection of pestivirus RNA by RT-qPCR targeting the NS3 gene in varying samples from pestivirus-inoculated and PBS-inoculated piglets.
(DOCX)
