# Supplementary material for: Identification of a Divergent Lineage Porcine Pestivirus in Nursing Piglets with Congenital Tremors and Reproduction of Disease following Experimental Inoculation
Source: PLoS One. 2016 Feb 24;11(2):e0150104. doi: 10.1371/journal.pone.0150104 (PMC4766193; doi:10.1371/journal.pone.0150104)
Supplement: S1 Table — The pestivirus was detected by RT-qPCR targeting the NS3 gene in the serum from a single sow that had farrowed piglets with congenital tremors. (DOCX) [file pone.0150104.s007.docx]

**S1 Table. Sow Serum RT-qPCR Results.** The pestivirus was detected by qRT-RCR targeting the NS3 gene in the serum from a single sow that had farrowed piglets with congenital tremors.

|  |  | ***Serum*** | |
| --- | --- | --- | --- |
| ***Farm*** | ***Sow ID*** | Cq | SQ |
| A | 55099 | U | 0.00E+00 |
|  | 56851 | U | 0.00E+00 |
|  | 54600 | U | 0.00E+00 |
|  | 57070 | U | 0.00E+00 |
|  | 55398 | U | 0.00E+00 |
| B | 1 | U | 0.00E+00 |
|  | 2 | 34.77 | 7.50E+02 |
|  | 3 | U | 0.00E+00 |
|  | 4 | U | 0.00E+00 |
|  | 5 | U | 0.00E+00 |
