# Supplementary material for: Identification of a Divergent Lineage Porcine Pestivirus in Nursing Piglets with Congenital Tremors and Reproduction of Disease following Experimental Inoculation
Source: PLoS One. 2016 Feb 24;11(2):e0150104. doi: 10.1371/journal.pone.0150104 (PMC4766193; doi:10.1371/journal.pone.0150104)
Supplement: S2 Table — a Detection of pestivirus RNA by RT-qPCR targeting the NS3 gene in varying samples from pestivirus-inoculated and PBS-inoculated piglets. (DOCX) [file pone.0150104.s008.docx]

**S2 Table. RT-qPCR Results by Piglet and Sample Type.^a^** Detection of pestivirus RNA by qRT-RCR targeting the NS3 gene in varying samples from pestivirus-inoculated and PBS-inoculated piglets.

| ***Sow ID/Inoculum/ Gestation Day^b^*** | ***Animal ID*** | **Serum** | **Nasal Swab** | **Feces** | **Terminal Serum** | **Cerebrum** | **Cerebellum** | **Spinal Cord** | **Brain Stem** | **CSF^c^** | **Kidney** | **M-LN^d^** | **TB-LN^e^** | **Thymus** | **Heart** | **Spleen** | **Umbilical Cord Blood^f^** | **Whole Blood** |
| --- | --- | --- | --- | --- | --- | --- | --- | --- | --- | --- | --- | --- | --- | --- | --- | --- | --- | --- |
| 2427/PBS/62 | 71 | U^g^ | U | U | U | U | U | U | U | U | U | U | U | U | U | U | ND^h^ | U |
|  | 72 | U | U | U | U | U | U | U | U | U | U | U | U | U | U | U | ND | U |
|  | 73 | U | U | U | U | U | U | U | U | U | U | U | U | U | U | U | ND | U |
|  | 74 | U | U | U | U | U | U | U | U | U | U | U | U | U | U | U | ND | U |
|  | 75 | U | U | U | U | U | U | U | U | U | U | U | U | U | U | U | ND | U |
|  | 124 | U | U | U | U | U | U | U | U | U | U | U | U | U | U | U | ND | U |
|  | 125 | U | U | U | U | U | U | U | U | U | U | U | U | U | U | U | ND | U |
| 4036/pestivirus/45 | 31 | U | U | U | 29.37 | U | 29.16 | 28.37 | 26.23 | ND | U | 25.59 | 26.69 | 26.15 | 25.99 | 24.06 | ND | 30.12 |
|  | 32 | 24.36 | U | U | 28.98 | 29.52 | 24.36 | 25.59 | 30.16 | 32.39 | 24.93 | 22.87 | 22.29 | 26.64 | 24.92 | 23.10 | ND | 24.07 |
|  | 33 | 24.06 | U | U | 21.74 | 29.33 | 21.33 | 27.68 | 34.50 | U | U | 25.12 | 21.85 | 25.24 | 26.70 | 23.04 | ND | 28.12 |
|  | 34 | U | U | 30.62 | 28.21 | U | U | U | U | U | U | U | U | U | U | U | ND | 27.77 |
|  | 35 | 28.13 | U | U | 29.27 | 30.22 | 20.02 | 25.13 | 26.78 | U | 26.80 | 26.56 | 22.38 | 26.28 | 26.66 | 23.73 | ND | 28.23 |
|  | 36 | U | U | 26.93 | 32.33 | 31.54 | 23.21 | 26.70 | 29.37 | 21.33 | U | 24.62 | 21.91 | 23.58 | 26.96 | U | ND | 27.53 |
|  | 37 | U | U | U | 28.28 | 28.46 | 28.38 | 26.47 | 29.89 | 23.44 | 25.88 | 27.00 | 25.84 | 24.63 | U | U | ND | 29.28 |
|  | 38 | U | U | U | 21.06 | 29.85 | 21.33 | 29.17 | 33.62 | ND | 25.43 | U | 24.88 | 24.12 | 25.47 | 23.20 | ND | 32.28 |
| 3992/pestivirus/45 | 40 | ND | ND | ND | 23.56 | 31.25 | 26.04 | 28.22 | 29.87 | 28.38 | 26.28 | 25.69 | U | 25.04 | U | 24.11 | ND | U |
|  | 41 | 23.08 | U | U | 27.28 | 33.75 | 30.35 | 28.24 | 31.97 | ND | 27.35 | 23.62 | 27.07 | 28.42 | 26.68 | 24.91 | 22.17 | 26.32 |
|  | 42 | U | U | U | 29.05 | 36.28 | 28.91 | 28.64 | 30.11 | 21.49 | U | 26.08 | U | U | U | 25.90 | ND | 29.12 |
|  | 43 | 27.30 | U | 30.75 | 25.15 | 31.42 | 24.05 | U | U | 22.95 | 25.09 | 24.67 | 22.88 | U | U | U | 23.04 | 29.11 |
|  | 44 | U | U | U | 28.42 | U | 23.40 | 29.08 | 30.02 | 23.07 | 31.39 | 24.23 | 27.81 | U | 31.35 | 28.00 | 26.46 | 27.05 |
|  | 45 | 24.74 | U | U | 25.43 | U | 29.56 | U | 31.13 | 25.99 | 27.65 | U | 25.40 | 30.13 | 28.36 | 25.28 | 26.64 | 27.42 |
|  | 46 | 24.37 | U | U | 30.48 | 30.21 | U | U | 33.00 | 25.42 | 25.69 | 30.55 | 36.47 | 25.67 | 24.45 | 23.47 | 25.88 | 24.83 |
|  | 47 | 21.80 | U | U | 29.16 | U | 28.52 | U | U | 26.92 | 26.03 | 26.93 | 27.28 | 25.10 | 27.88 | 23.94 | U | 27.15 |
|  | 48 | 24.28 | U | U | 27.30 | U | 26.20 | U | 32.93 | 27.73 | 27.63 | 29.25 | 27.54 | 33.00 | 31.38 | 25.93 | U | 28.29 |
| 3661/pestivirus/62 | 94 | 25.85 | U | U | 20.20 | 31.12 | 22.50 | 28.01 | 29.00 | ND | 25.56 | 23.20 | 23.65 | 25.32 | 25.00 | 23.72 | ND | ND |
|  | 95 | U | U | U | U | U | U | U | U | ND | U | U | U | U | U | U | ND | ND |
|  | 96 | U | U | U | 21.07 | 29.86 | 27.01 | 31.12 | 28.69 | ND | 25.43 | 24.35 | 24.10 | 26.48 | 25.92 | 24.61 | ND | ND |
|  | 97 | 24.67 | U | U | 22.73 | 27.19 | 29.45 | 28.79 | 27.37 | ND | 27.03 | 22.50 | 23.79 | 24.91 | 25.51 | 24.77 | ND | ND |
|  | 98 | U | U | U | 22.64 | 28.93 | 31.47 | 29.57 | 30.89 | ND | 27.04 | 23.98 | 24.14 | 26.20 | 25.63 | 24.59 | ND | ND |
|  | 99 | U | U | U | 20.36 | 28.93 | 31.47 | 29.57 | 30.89 | ND | 27.59 | 22.52 | 22.06 | 23.77 | 23.71 | 23.20 | ND | ND |
|  | 100 | 23.87 | U | U | 21.38 | U | 26.33 | 29.32 | 31.69 | ND | 26.61 | 22.93 | 23.10 | 23.56 | 23.78 | 24.96 | ND | ND |
| 3500/pestivirus/62 | 89 | U | 22.07 | 26.22 | 28.39 | 30.77 | 25.41 | 27.05 | 27.98 | 27.05 | 26.16 | 24.61 | 31.41 | 28.33 | 24.81 | 24.17 | ND | 30.25 |
|  | 90 | U | U | U | 25.24 | 27.60 | 25.16 | 26.09 | 28.04 | ND | 27.38 | 25.74 | 27.73 | 27.81 | 28.96 | 26.33 | ND | 31.48 |
|  | 111 | U | 28.86 | 28.18 | 31.00 | 29.64 | 26.00 | 26.02 | 30.29 | 27.40 | 26.27 | 23.28 | 25.55 | 22.44 | 25.02 | 24.40 | ND | 27.27 |
|  | 112 | 22.52 | U | U | ND | ND | ND | ND | ND | ND | ND | ND | ND | ND | ND | ND | ND | ND |
|  | 113 | 25.90 | U | U | 29.21 | 26.39 | 28.24 | 26.22 | 27.08 | ND | 26.56 | 27.48 | 25.56 | 26.03 | 26.57 | 28.46 | ND | U |
|  | 116 | U | 29.10 | U | 30.74 | 30.45 | 27.43 | 27.21 | 28.72 | 26.21 | 26.09 | 27.06 | 24.50 | 27.75 | 26.13 | 24.27 | ND | U |
|  | 117 | 21.99 | U | U | 30.48 | 24.85 | 28.83 | 26.35 | 26.58 | 24.67 | 26.03 | 25.27 | 24.48 | 27.60 | 24.65 | 24.35 | ND | U |
|  | 118 | 21.97 | 32.25 | U | 22.47 | 27.66 | 31.88 | 28.07 | 26.79 | 26.20 | 26.14 | 24.02 | 26.15 | 25.78 | 25.63 | 24.90 | ND | U |
|  | 121 | 22.43 | 31.08 | U | 28.62 | 27.42 | 26.18 | 27.31 | 29.31 | 25.97 | 28.62 | 26.19 | 25.56 | 31.15 | 34.52 | 28.20 | ND | U |
|  | 122 | 20.85 | U | 27.46 | 30.14 | 30.39 | 24.31 | 25.88 | 28.41 | 24.07 | 26.97 | 23.48 | 22.98 | 27.68 | 25.90 | 23.94 | ND | U |
|  | 123 | 20.65 | U | 25.89 | 29.39 | 28.37 | 21.74 | 26.61 | 28.62 | 24.14 | 24.52 | 26.46 | 24.51 | 23.28 | 26.33 | 24.65 | ND | U |
| 4023/pestivirus/62 | 114 | 21.40 | 26.60 | 24.00 | U | U | U | 26.34 | 30.48 | 30.88 | 25.47 | 24.14 | 24.50 | 30.60 | 28.64 | 29.37 | ND | U |
|  | 115 | 22.00 | 21.06 | 26.63 | U | U | U | 28.36 | 30.80 | 22.75 | 30.29 | 29.35 | 23.64 | 25.19 | 32.41 | 25.18 | ND | U |
|  | 119 | 21.82 | 21.51 | 28.31 | U | 24.58 | 23.65 | U | 20.80 | 22.36 | 26.17 | 24.70 | 33.74 | 29.47 | 27.81 | 26.01 | ND | U |
|  | 120 | 21.12 | 24.48 | 23.93 | U | 24.74 | 26.22 | 31.73 | 25.64 | 27.06 | 26.47 | 25.27 | 25.37 | 27.24 | 28.80 | 25.29 | ND | U |
|  | 130 | 22.22 | 23.62 | 25.40 | U | 26.12 | 29.00 | 32.47 | 24.23 | 26.12 | 26.26 | 24.91 | 26.35 | 27.66 | 26.27 | 25.90 | ND | U |
|  | 131 | 22.43 | 22.43 | 27.05 | U | 24.34 | 22.72 | 24.83 | 26.58 | 24.91 | 30.24 | 30.10 | 28.52 | 30.92 | 25.12 | 27.14 | ND | U |
|  | 132 | 21.36 | 24.31 | 26.00 | 31.86 | 22.78 | 24.08 | 26.35 | 25.58 | 22.95 | 27.16 | 24.03 | 23.51 | 28.39 | 28.80 | 28.42 | ND | U |

^a^Samples with cycle threshold (Cq) values >36 were considered negative.

^b^Day of gestation at time of inoculation.

^c^CSF = Cerebrospinal fluid.

^d^M-LN = Mesenteric lymph node.

^e^TB-LN = Tracheobronchial lymph node.

^f^Cord blood = Umbilical cord blood.

^g^U indicates “undetected” following 40 cycles.

^h^ND = Not done.
